# A Novel Monoclonal Antibody 1D2 That Broadly Inhibits Clinically Important *Aspergillus* Species

**DOI:** 10.3390/jof8090960

**Published:** 2022-09-14

**Authors:** Xihua Lian, Amy Scott-Thomas, John G. Lewis, Madhav Bhatia, Stephen T. Chambers

**Affiliations:** 1 Department of Pathology and Biomedical Science, University of Otago, Christchurch 8140, New Zealand; 2 Department of Medical Imaging, The Second Clinical Medical School, Fujian Medical University, Quanzhou 362000, China; 3 Canterbury Health Laboratories, Christchurch 8140, New Zealand

**Keywords:** monoclonal antibody, treatment, *Aspergillus* infection, invasive aspergillosis

## Abstract

*Aspergillus fumigatus* is a ubiquitous airborne fungus, is the predominant cause (>90%) of invasive aspergillosis (IA) in immunosuppressed patients and has a high mortality. New approaches to prevention and treatment are needed because of the poor efficacy, toxicity and side effects of the current anti-*Aspergillus* drugs on patients. Thus, we aim to explore a new avenue to combat *Aspergillus* infection by using a novel monoclonal antibody (mAb) 1D2 against a glycoprotein on the cell wall of *Aspergillus*. The ability of this mAb to inhibit attachment, germination, and growth of *Aspergillus* conidia and hyphae in vitro were examined. A dose-dependent growth inhibition of *Aspergillus* conidia in the presence of mAb 1D2 was found. The mAb 1D2 inhibited attachment of *Aspergillus* conidia to an untreated slide surface and fibronectin-treated surface compared to an unrelated mAb 6B10. When conidia were exposed to 1D2 concomitantly with inoculation into culture media, the mAb prevented the swelling and germination of conidia. This inhibitory ability of 1D2 was less apparent if it was added two hours after inoculation. Damage to hyphae was also observed when 1D2 was added to *Aspergillus* hyphae that had been incubated in media overnight. These in vitro results indicate that mAb 1D2 broadly inhibits clinically important *Aspergillus* species and has a promising therapeutic effect both as prophylaxis to inhibit an *Aspergillus* infection as well as a treatment.

## 1. Introduction

*Aspergillus fumigatus* is an airborne fungus found ubiquitously in our surrounding environment. *A. fumigatus* is the most common mould, causing severe fungal infections ranging from *Aspergillus* allergic diseases to invasive aspergillosis (IA). IA is an opportunistic, potentially lethal and systemic infectious disease mainly occurring in immunosuppressed patients [1]. The increasing number of patients being treated with immune suppressive regimens has led to increasing numbers of susceptible patients and an increase in case numbers of IA. Worldwide, there are more than 200,000 fatal infections caused by IA annually [2,3]. Prevention, a timely diagnosis and effective anti-*Aspergillus* therapy are needed to improve IA prognosis.

Currently, three categories of antifungal drugs including triazoles, amphotericin B and echinocandins are used to principally treat *Aspergillus* infection [4,5,6]. However, there is increasing concern about the inevitable toxicity and side effects on patients when they receive antifungal therapy for long periods. In addition, antibiotic overuse and resistance, caused by inappropriate anti-*Aspergillus* prevention or drug usage, remains a major challenge [3]. Despite the availability of these agents, there is a high case-fatality rate of IA in immunodeficient patients which is reported to be as high as 90% in some studies [2]. While surgery may be an approach for those who have focal infection, it is not indicated for many severely immunosuppressed patients due to postoperative complications [7]. The difficulty in managing an *Aspergillus* infection has spurred interest in pursuing a new, rapid acting, effective and specific anti-*Aspergillus* therapeutic avenue.

A monoclonal antibody (mAb) is produced by a single B-lymphocyte clone with highly specific binding to the matching antigen. In the past decades, mAbs have been rapidly developed and employed in treating various diseases, such as malignant tumours [8], hyperlipidaemia [9], migraine [10] and infectious diseases [11] including influenza [12] and COVID-19 [13,14]. However, little attention has been devoted to the use of mAb in managing *Aspergillus* infection. 

**Figure 1:**
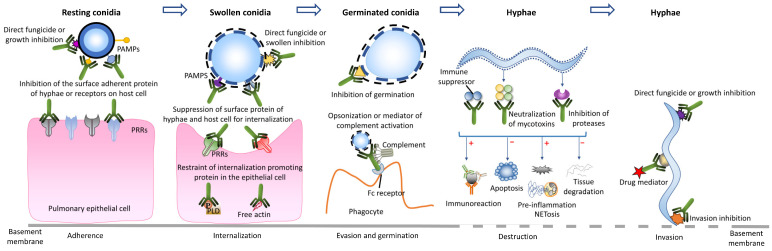
Monoclonal antibody-based modes to suppress or eliminate *Aspergillus.* The interference of monoclonal antibody in the process of adherence, internalization of *Aspergillus* conidia, or the process of germination, destruction and invasion of *Aspergillus* hyphae may be a potential treatment target to protect the host. Adapted from Lian X. et al. [23].

*Aspergillus* infection follows a complicated sequence of events including conidia adherence and internalisation that is followed by hyphal invasion and the release of proteases and toxins. Thus, a mAb that is specific to either a cellular or extracellular antigen may play a critical role in preventing or treating *Aspergillus* infection. For example, mAbs involved in adherence suppression [15,16], opsonization [17], neutralization of toxins or enzymes [18], complement activation and directing fungicidal activity [15,19,20] (Figure 1) may downregulate the pathogenicity, invasion and dissemination of *Aspergillus* [21,22] or induce pathogen destruction [23]. 

In this study, we aimed to determine the therapeutic potential of an antibody-based approach against *Aspergillus* by determining the effects of a novel mAb 1D2 on key steps needed in the pathogenesis of infection. The mAb 1D2 has high sensitivity and specificity to a glycoprotein on the cell wall of *A. fumigatus* and *A. flavus* [24].

## 2. Materials and Methods

### 2.1. Fungal Strains and Growth Conditions

*A. fumigatus* (AF 293) and *Aspergillus flavus* (NRRL 3357) were obtained from the American Type Culture Collection (ATCC). Two *A. fumigatus*, one *Aspergillus* *niger* and one *Aspergillus* *terreus*, clinical strains were obtained from the Canterbury Health Laboratories, Christchurch, New Zealand. All microorganisms were cultured on Sabouraud dextrose agar (SDA) plates or in Sabouraud dextrose (SD) liquid media at 37 °C.

### 2.2. Management of Aspergillus Conidia Suspension

The preparation of *Aspergillus* conidia suspension was similar to our previous study with only minor changes [24]. Various *Aspergillus* species were streaked on SDA plates and cultured for five to seven days; a single colony of conidia was sub-cultured on a new SDA plate until adequate amounts of conidia were visualised. Conidia were collected by washing the plate with 0.1% Tween 20 in phosphate-buffered saline (PBST) and filtered through an autoclaved Miracloth (Merck, Darmstadt, Germany). The conidial suspension was centrifuged (5000× *g*) and washed three times with PBST between spins. After discarding the supernatant, the conidial precipitate was resuspended with PBS and adjusted to 1 × 10^5^ conidia/mL, 2 × 10^5^ conidia/mL, 5 × 10^5^ conidia/mL, 2 × 10^6^ conidia/mL and 2 × 10^8^ conidia/mL, respectively, using a haemocytometer and stored at 4 °C.

### 2.3. The Production and Purification of mAb 1D2 

The production of mAb 1D2 has been described previously [24]. 1D2 was purified by a mouse IgM purification resin column (LT-145, LigaTrap, Raleigh, NC, USA) according to the manufacturer’s instructions. Antibody concentration was determined by a UV–visible spectrophotometer at 280 nm and stored at −20 °C. The unrelated control IgM antibody (6B10) was similarly purified. The various working concentrations of monoclonal antibodies were diluted in SD broth.

### 2.4. In Vitro Biological Activity of mAb 1D2

#### 2.4.1. Growth Inhibition Assay

The growth inhibition activity of mAb 1D2 against different *Aspergillus* species was determined as defined by Magliani [25] with some amendments. An aliquot of 10 µL of *Aspergillus* conidia suspension (1 × 10^5^ conidia/mL) was added to a sterile 96-well plate containing 100 µL of purified mAb 1D2 at various working concentrations (0.78125, 1.5625, 3.125, 6.25, 12.5, 25 and 50 µg/mL) in SD broth. PBS and the unrelated mouse IgM 6B10, at similar concentrations in the SD broth, were used as controls [26]. After incubation for 18 h at 37 °C, the *Aspergillus* cell suspension was spread on SDA plates (50 μL) and incubated at 37 °C for 24 to 48 h to measure the colony-forming units (CFUs). In addition, to assess whether growth inhibition was caused by antibody binding to the conidia, we centrifuged the samples after overnight incubation and washed 5 times to remove any un-bound antibody in the media but were otherwise treated the same. Finally, the growth inhibition percentage was calculated as follows.

$$\mathrm{Growth}\;\mathrm{inhibition}\;\mathrm{percentage}=\frac{\mathrm{CFUs}\;\left(\mathrm{control}\right)-\mathrm{CFUs}\;\left(\mathrm{experimental}\right)}{\mathrm{CFUs}\;\left(\mathrm{control}\right)}\times 100$$

From this assay, the concentration of mAb 1D2 required for 50% of the growth reduction of various *Aspergillus* species compared to 6B10 control group was determined. All groups in this study were performed in triplicate. 

#### 2.4.2. Attachment Inhibition Assay

Attachment inhibition effect of mAb 1D2 against various *Aspergillus* species was determined as described previously by Gravelat et al. [27] with some modifications. Assays were performed in 8-well chamber slides (Nunc™ Lab-Tek™ II Chamber Slide™ System, Thermo Fisher Scientific, Waltham, MA, USA) with or without fibronectin coating. Fibronectin wells were coated with 200 µL of fibronectin at 10 µg/mL and incubated at room temperature (RT) for 4 h. Fibronectin was then aspirated; the wells rinsed 3 times with PBS and airdried for 1 h at RT. Thereafter, 200 µL of purified antibody 1D2 (100 µg/mL) diluted in SD broth was added to each well which contained 100 µL of *Aspergillus* conidia suspension (2 × 10^5^ conidia/mL) and incubated for 8 h at RT on the bench without shaking. The final concentration of 1D2 was 66 µg/mL. PBS and mAb 6B10 were used as controls respectively. Bovine serum albumin (BSA) 5% in PBS was also used as a “blocking” control to detect any non-specific protein binding. The wells were washed three times with PBS to remove non-adhesive conidia. The number of conidia adhered to the untreated surface or fibronectin coated surface were observed using an inverted phase-contrast microscope (Olympus, Tokyo, Japan). After visualisation, the attached conidia were removed by washing the wells with PBS containing 0.1% TritonX-100. The washed *Aspergillus* conidial suspension was mixed well; 50 μL was spread onto SDA plates and incubated at 37 °C for 24 to 48 h to calculate the CFUs. The attachment inhibition percentage was calculated as follows.

$$\mathrm{Attachment}\;\mathrm{inhibition}\;\mathrm{percentage}=\frac{\mathrm{CFUs}\;\left(\mathrm{control}\right)-\mathrm{CFUs}\;\left(\mathrm{experimental}\right)}{\mathrm{CFUs}\;\left(\mathrm{control}\right)}\times 100$$

#### 2.4.3. Swelling and Germination Inhibition and Antibody Intervention Assay

The germination assay has been described previously and was amended slightly [28]. A 20 µL aliquot of *Aspergillus* conidia suspension (2 × 10^8^ conidia/mL) and 100 µL of mAb 1D2 (1000 µg/mL) were incubated in 1000 µL of SD media at 37 °C shaking at 200 rpm/h. The final concentration of 1D2 was 89 µg/mL. At regular intervals of 1 h for 24 h, and then at 24 h, 10 µL aliquots were removed and swollen or germinated conidia were counted using a haemocytometer. The swollen or germination percentage of each sample was calculated as:

$$\mathrm{Swollen}\;\mathrm{or}\;\mathrm{germination}\;\mathrm{percentage}=\frac{\mathrm{swollen}\;\mathrm{or}\;\mathrm{germinated}\;\mathrm{conidia}\;\mathrm{number}}{\mathrm{Total}\;\mathrm{counted}\;\mathrm{conidia}\;\mathrm{number}}\times 100$$

To determine the effect of antibody on swelling and germination of *Aspergillus* conidia at different growth time points, 20 µL aliquots of a suspension of *Aspergillus* conidia (2 × 10^8^ conidia/mL) were added to 1000 µL of SD media. The monoclonal antibody 1D2 (100 µL of 1000 µg/mL) was then added to each tube every hour from the beginning of the incubation until the tenth hour. After the last addition of 1D2 to the 11th tube, it was incubated for 1 more hour. Samples (10 µL) were then taken from each tube and examined microscopically. PBS and mAb 6B10 were used as a negative and isotype control, respectively. The swollen or germination inhibition percentage was calculated as follows:

$$\mathrm{Inhibition}\;\mathrm{percentage}=\frac{\mathrm{swollen}/\mathrm{germinated}\;\mathrm{conidia}\;\left(\mathrm{control}\right)-\mathrm{swollen}/\mathrm{germinated}\;\mathrm{conidia}\;\left(\mathrm{experimental}\right)}{\mathrm{swollen}/\mathrm{germinated}\;\mathrm{conidia}\;\left(\mathrm{control}\right)}\times 100$$

Finally, the remaining culture media containing *Aspergillus* pellets of each group were centrifuged at 5000× *g* for 5 min. Without disturbing the pellets, 500 μL of supernatant was discarded, and the remaining media and hyphae were disrupted by sonication at 4 °C. The broken *Aspergillus* hyphal mixture was mixed using a pipette, and then, 100 μL of each sample was aliquoted into a 96-well microtiter plate and the absorbance at 600 nm (A_600_) was read in a microplate spectrophotometer (Thermo Fisher Scientific, Waltham, MA, USA).

To determine whether the swelling and germination of *Aspergillus* was completely inhibited or partly inhibited, a secondary test was set up. The mAb 1D2 was added to the culture after *Aspergillus* conidia had been incubated for 6 h, and the *Aspergillus* morphology changes were observed under a microscope at the 6th, 10th, 24th, 32nd and 72nd hour of culture. 

#### 2.4.4. The Effect of mAb 1D2 on Hyphal Metabolic Activity by XTT Assay 

The 2,3-Bis-(2-Methoxy-4-Nitro-5-Sulfophenyl)-2H-Tetrazolium-5-Carboxanilide (XTT) (Invitrogen, Waltham, Massachusetts, USA) hyphae damage assay was completed in accordance with the commercial kit instruction and previous studies [29,30] with minor modifications. Briefly, 10 µL of *Aspergillus* conidia suspension (5 × 10^5^ conidia/mL) was added to 100 µL of SD media, seeded to a 96-well plate (Corning, NY, USA) and incubated overnight at 37 °C to form hyphae. After overnight incubation, the culture media were removed, and the hyphae were washed with sterile PBS. To determine the optimum mAb 1D2 concentration for future work, 100 µL of mAb 1D2 at varying concentrations (6.25, 12.5, 25, 50, 100, 200, and 400 µg/mL) were then added to each well and incubated for a further 24 h. After incubation, the supernatant was discarded, and the hyphae were washed three times with sterile PBS. The mAb 6B10 was used as an isotype control alongside wells with *Aspergillus* hyphae without mAb which were set as a positive control. XTT working solution was prepared immediately before the assay. An aliquot of 70 μL of the XTT working solution was directly added to each well containing 100 µL of PBS and incubated for 4 h at 37 °C. After incubation, the absorbance was read at 450 nm (A_450_) on a microplate reader (Thermo Fisher Scientific, Waltham, Massachusetts, USA). The percent of hyphal metabolic activity abnormality caused by mAb 1D2 was calculated as follows.

$$\mathrm{Hyphal}\;\mathrm{metabolic}\;\mathrm{activity}\;\mathrm{abnormality}\;\mathrm{percentage}=\frac{A450\;\left(\mathrm{control}\right)-A450\;\left(\mathrm{experimental}\right)}{A450\;\left(\mathrm{control}\right)}\times 100$$

### 2.5. Statistical Analysis

Statistical analysing in this study was completed by SPSS software (version 21.0, IBM Analytics, New York, NY, USA) and GraphPad software (version 9, Prism, San Diego, CA, USA). Continuous variables were demonstrated as (mean ± standard deviation (SD)) if the data were normally distributed or (median (interquartile range)) if the data were not normally distributed. Data were analysed by independent samples *t* test or one-way ANOVA with post hoc Dunnett T3 test. A *p* value < 0.05 was considered statistical significance.

## 3. Results

### 3.1. In Vitro Growth Inhibition Activity of mAb 1D2 against Different Aspergillus Species

**Figure 2:**
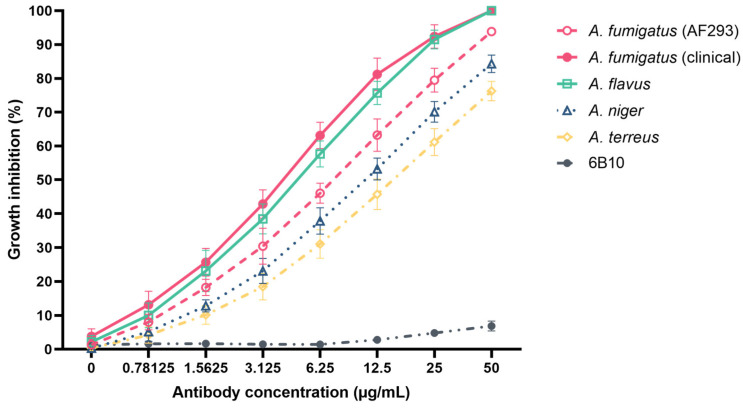
In vitro growth inhibition activity of mAb 1D2 with various concentrations against *A. fumigatus*, *A. flavus*, *A. niger* and *A. terreus*. Compared to the control mAb 6B10, mAb 1D2 showed significant growth inhibition on *A. fumigatus* (two clinical strains), *A. flavus* and *A. fumigatus* (AF 293), followed by *A. niger* and *A. terreus*. With the increase in antibody concentration, the growth inhibition effect of 1D2 steadily increased.

Compared to mAb 6B10, mAb 1D2 showed significant growth inhibition, as determined by CFUs, on *A. fumigatus* (including the ATCC strain and two clinical strains), *A. flavus*, *A. niger* and *A. terreus*, respectively, when the antibody concentration was more than 1.5625 μg/mL (*p* < 0.001) (Figure 2). Increasing antibody concentration correlated with increase in growth inhibition. All *Aspergillus* species growth were significantly inhibited by 1D2 when antibody concentrations were 50 μg/mL. Moreover, the lowest concentrations of 1D2 that caused less than 50% growth of *A. fumigatus*, *A. flavus*, *A. niger* and *A. terreus* were 7.45 ± 0.21, 4.68 ± 0.93, 10.49 ± 1.58 and 14.72 ± 2.74 μg/mL, respectively. Additionally, for the centrifuged samples, we found when compared to the mAb 6B10 group, mAb 1D2 significantly inhibits growth, shown by a decrease in CFUs. This also indicates that the mAb 1D2 binds to the early swollen conidia to suppress growth.

### 3.2. Attachment Inhibition Assay

**Figure 3:**
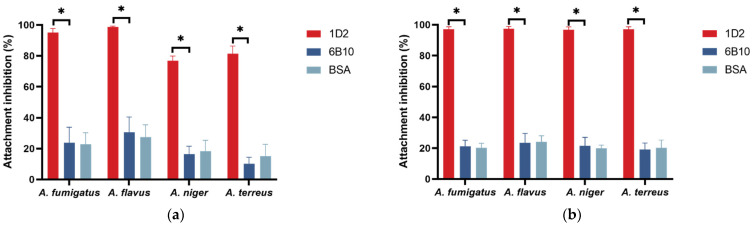
In vitro attachment inhibition of mAb 1D2 against *A. fumigatus*, *A. flavus*, *A. niger* and *A. terreus*. Compared to an unrelated mAb 6B10, mAb 1D2 (20 μg) showed statistically significant adhesive inhibition on *A. fumigatus*, *A. flavus*, *A. niger* and *A. terreus* (2 × 10^4^ conidia), respectively, on untreated slide surfaces (**a**) and on fibronectin-treated slide surfaces (**b**). There was no difference between the mAb 6B10 control group and BSA control group for all *Aspergillus* species. *: *p* < 0.001 versus 6B10 group.

The ability of mAb 1D2 to inhibit attachment was examined for *A. fumigatus*, *A. flavus*, *A. niger* and *A. terreus* on the untreated slide surface (Figure 3a) and fibronectin-coated surface (Figure 3b). Compared to IgM 6B10, mAb 1D2 significantly reduced the adherence of *A. fumigatus* (23.71 ± 10.10% vs. 95.08 ± 2.54%, *p* < 0.001), *A. flavus* (30.58 ± 9.89% vs. 98.60 ± 0.66%, *p* < 0.001), *A. niger* (16.31 ± 5.20% vs. 76.82 ± 3.09%, *p* < 0.001) and *A. terreus* (10.20 ± 4.18% vs. 81.46 ± 4.90%, *p* < 0.001) on the untreated slide surface (Figure 3a). To confirm the anti-adherence effect of mAb 1D2, fibronectin-coated chamber slides were seeded with conidia from the four *Aspergillus* species. This showed that mAb 1D2 grossly inhibited the adhesion of *A. fumigatus* (21.07 ± 4.05% vs. 97.24 ± 1.47%, *p* < 0.001), *A. flavus* (23.50 ± 6.02% vs. 97.46 ± 1.51%, *p* < 0.001), *A. niger* (21.46 ± 5.59% vs. 96.88 ± 1.81%, *p* < 0.001) and *A. terreus* (19.05 ± 4.28% vs. 97.08 ± 1.62%, *p* < 0.001) on fibronectin compared to the mAb 6B10 (Figure 3b). There was no significant difference of the attachment inhibitory percentage between the mAb 6B10 group and BSA control group for the four *Aspergillus* species (*p* > 0.05).

### 3.3. Swelling and Germination Inhibition Assay

#### 3.3.1. Swelling and Germination Rate of mAb 1D2 against Different *Aspergillus* with Increasing Incubation Times

**Figure 4:**
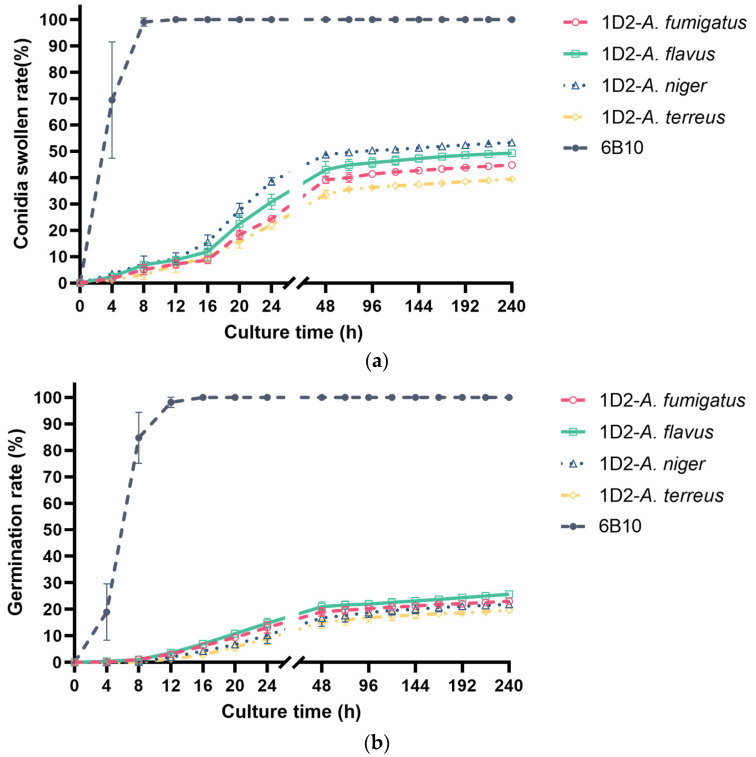
Swelling and germination rate of different *Aspergillus* conidia against mAb 1D2. The mAb 1D2 markedly delayed both the swelling (**a**) and germination process (**b**) (*p* < 0.001) for *A. fumigatus*, *A. flavus*, *A. niger* and *A. terreus*. While there were slight conidia swelling prior to 16 h, swelling and germination continued and reached a stationary phase after 48 h. Conversely, the control mAb 6B10 did not show apparent interference on conidial swelling (**a**) or germination (**b**). The resting conidia started to swell within the first two hours of incubation and were almost completely swollen when cultured for 8 h (**a**). The swollen conidia activated germination when incubated for 4 h, and most of them germinated after 10 h (**b**).

**Figure 5:**
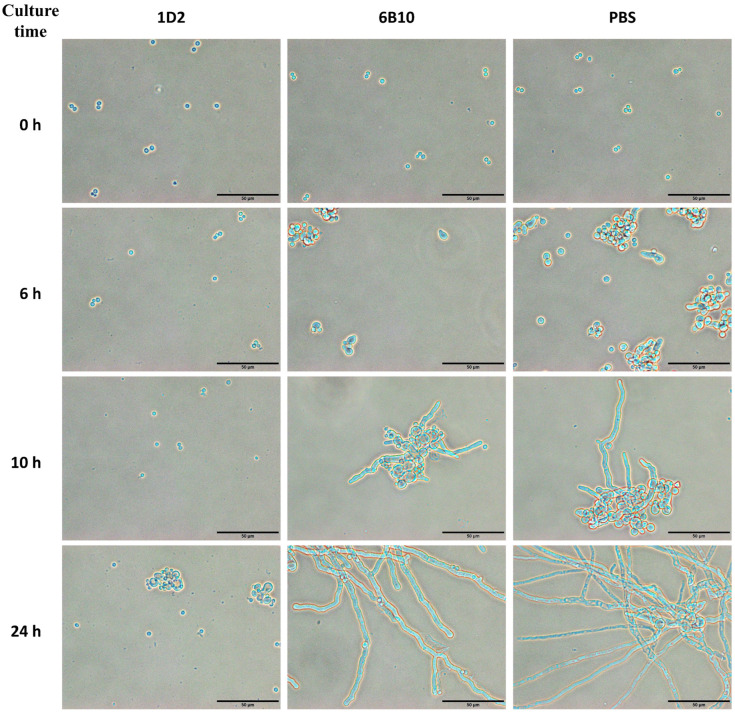
*Aspergillus* morphology at different times after treatment with mAb 1D2, 6B10 and PBS. MAb 1D2, compared to the control mAb 6B10, extensively delayed conidial swelling and germination. There were occasional swollen *Aspergillus* conidia or germ tubes within 10 h of culture and evidence of swollen and early germ tubes only after incubation for 24 h. Compared to the PBS control group, the mAb 6B10 did not show significant differences in conidia swelling and germination as the incubation period increased. Resting conidia in PBS and mAb 6B10 groups started swelling within 1 to 2 h in SD media and germinating after 4 h of culture. After 24 h of incubation, most of the conidia in these two groups had germinated to long branched mature hyphae with septum. Scale bar represents 50 μm.

We added mAb 1D2 to *Aspergillus* culture media to investigate the inhibitory effects of the mAb on *Aspergillus* conidial swelling and germination. Most of the resting conidia started to swell within the first and second hour of incubation, and almost all were swollen by 8 h (Figure 4a and Figure 5). The swollen conidia activated germination in most conidia by the fourth hour of incubation, and most germinated after 10 h (Figure 4b and Figure 5). MAb 1D2 delayed both the swelling and germination process of the conidia compared with controls and reached a plateau after incubation for 48 h. Conversely, compared to the PBS control, the mAb 6B10 showed no significant interference on either swelling or germination of *A. fumigatus*, *A. flavus*, *A. niger* and *A. terreus* (*p* > 0.05).

There were low numbers of swelling conidia in mAb 1D2 treated groups before 16 h of incubation, however after this time point, conidia became swollen, although this did not develop further in any species (Figure 4a and Figure 5). After 48 h of incubation with mAb 1D2, swelling was most inhibited in *A. niger* conidia (swollen rate 48.75 ± 1.26%), followed by *A. flavus* (swollen rate 43.02 ± 3.13%), *A. fumigatus* (swollen rate 39.21 ± 1.37%) and *A. terreus* (swollen rate 33.62 ± 1.56%). The swelling rates of all *Aspergillus* species tested in mAb 1D2 groups were significantly lower than that in mAb 6B10 groups (*p* < 0.001).

Germination of the various *Aspergillus* species was curbed by mAb 1D2 up to 20 h of incubation, but germ tubes were observed more readily after that. MAb 1D2 reached maximum inhibition after 48 h incubation against *A. flavus* (germination rate 20.95 ± 1.86%), *A. fumigatus* (germination rate 19.03 ± 1.59%), *A. niger* (germination rate 16.72 ± 3.18%) and *A. terreus* (germination rate 15.14 ± 2.42%) (Figure 4b and Figure 5). The germination rates of all *Aspergillus* species tested in mAb 1D2 groups were significantly lower than that in mAb 6B10 groups (*p* < 0.001). 

#### 3.3.2. The Effect of the Time of Conidia Exposure to mAb 1D2 on the Swelling and Germination of Different *Aspergillus*

**Figure 6:**
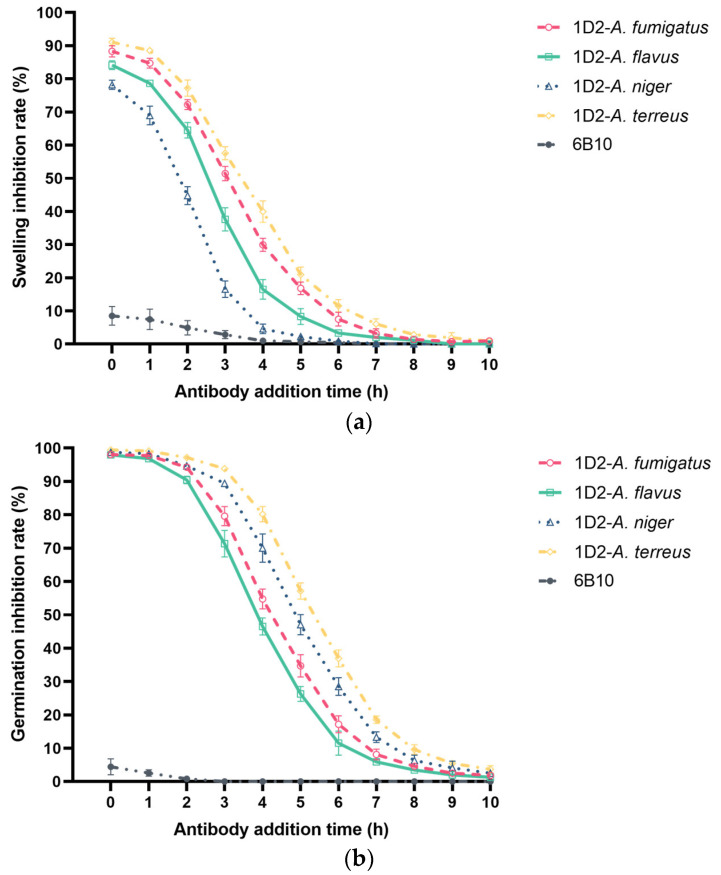

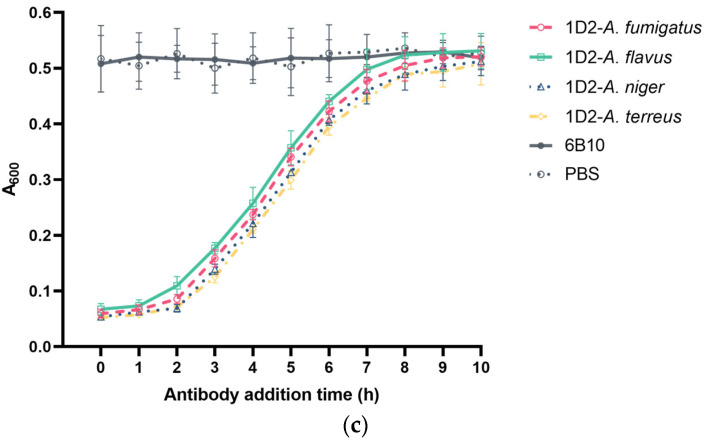
Effect of 1D2 (100 μg) intervention on *Aspergillus* conidial swelling and germination as a function of time. MAb 1D2 treatment resulted in significant inhibition of *Aspergillus* swelling (**a**) when added together with the conidia (0 h). The swelling inhibition ability against all *Aspergillus* species was downregulated markedly and reached a lower plateau after the sixth hour. In addition, mAb 1D2 showed significant inhibition capability on *Aspergillus* germination when it was added at the first two hours of culture (**b**), and the inhibitory ability dropped sharply between the third and seventh hour of culture. These inhibitory effects at different time points result in significantly lower absorbance (**c**) in the first two hours, which increased dramatically between the third and the seventh hour, and then finally hit stationary phase after seven hours of incubation. A_600_: absorbance at 600 nm.

We observed the swelling and germination rates of each sample together after 11 h incubation. Compared to mAb 1D2, 6B10 had little effect on the swelling and germination of *Aspergillus* and showed a significantly lower inhibition rate (*p* < 0.001). Conversely, mAb 1D2 inhibited swelling of *Aspergillus* conidia when added together (0 h) and inoculated into growth media. These inhibition rates were more than 80% for *A. terreus*, *A. fumigatus*, *A. flavus*, and more than 75% for *A. niger* (Figure 6a). Inhibition of swelling of conidia of all *Aspergillus* species declined sharply, as the time between inoculation of conidia into the growth medium and addition of mAb increased and was lost by 8 h. MAb 1D2 showed significant inhibition capability on *Aspergillus* germination when it was added during the first two hours of culture (germination inhibition rates were all more than 90% for all *Aspergillus* species) (Figure 6b). The percent dropped progressively when the antibody was added between the third hour and the seventh hour of incubation. 

In parallel, the addition of mAb 1D2 attenuated *Aspergillus* growth and therefore the formation of hyphal aggregates in the media for all four species, leading to lower absorbances compared to the mAb 6B10 group (*p* < 0.05, Figure 6c). When mAb 1D2 was added in the early 2 h of incubation, absorbances were extremely low, indicating inhibition of mAb 1D2 on *Aspergillus* germination. If added between the third and seventh hour, the turbidity of the culture media increased proportionally to growth, leading to the decreasing absorbance difference between 1D2 group and the 6B10 group (*p* < 0.05, Figure 6c). Addition of mAb 1D2 after 7 h did not inhibit germination, and the turbidities from *Aspergillus* aggregation did not show statistical significance to the mAb 6B10 group since it showed no effect on the formation of mycelia when compared to the PBS group (*p* > 0.05, Figure 6c).

**Figure 7:**
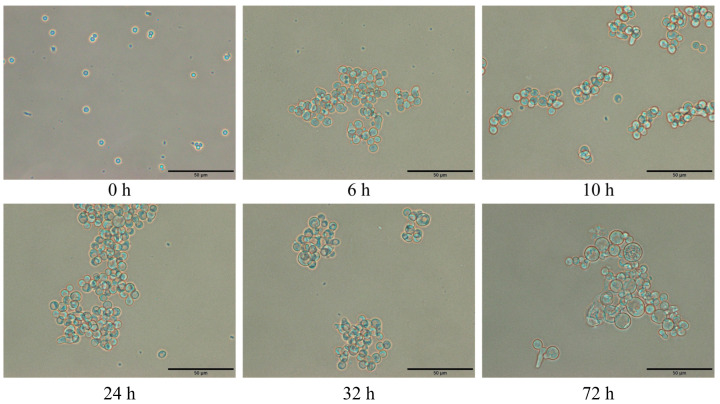
*Aspergillus* culture showing morphology changes when treated with mAb 1D2 after culturing for 6 h. After *Aspergillus* conidia were cultured for 6 h, most of the *Aspergillus* conidia were swollen, and some showed early germ tubes. When mAb 1D2 was added (6 h), conidia morphology changes were observed at the 10th, 24th, 32nd and 72nd hour of incubation. There was no significant development in germ tubes, but conidia became more swollen by 72 h. Scale bar represents 50 μm.

Conidia that had been cultured for 6 h with the subsequent addition of 1D2 were observed over time and showed interference by 1D2 (Figure 7). Most of the *Aspergillus* conidia were swollen, and some early germ tubes were apparent when cultured for 6 h before the addition of 1D2; however, the conidia failed to develop germ tubes or hyphae over the next 72 h, but the conidia became progressively more swollen (*p* < 0.001). The diameters of swollen conidia over time are demonstrated in Table 1. 

### 3.4. The Effect of mAb 1D2 on Hyphal Metabolic Activity by XTT Assay

**Figure 8:**
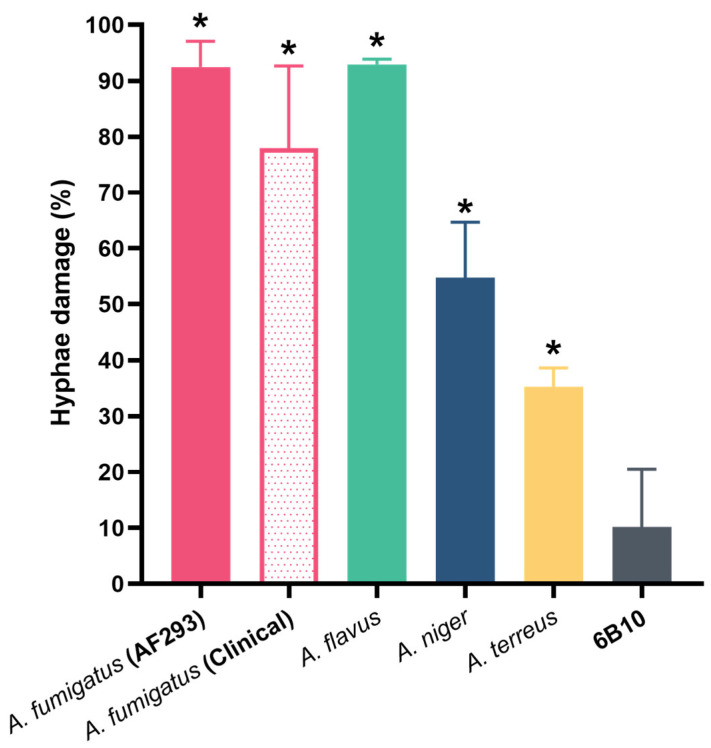
MAb 1D2 induced hyphae damage in various *Aspergillus* species. MAb 1D2 showed statistically significant effects on hyphal metabolic activity of *A. fumigatus* (AF 293), *A. fumigatus* (two clinical strains), *A. flavus*, *A. niger* and *A. terreus*, respectively. *: *p* < 0.001 versus 6B10 group.

The XTT assay is designed to measure metabolic activity as a marker of viability of cells. An initial optimization assay was performed to determine the metabolic activity inhibition of varying mAb concentrations on *A. fumigatus* AF 293 hyphae that developed after incubation of conidia overnight. This result indicated that a mAb 1D2 concentration of 50 μg/mL had an optimal effect on hyphal metabolism of this *Aspergillus* strain. Thus, this concentration was utilized to examine the hyphae metabolic activity against all *Aspergillus* species, and it showed inhibition to the hyphae of *A. fumigatus* (including ATCC strain and two clinical strains), *A. flavus*, *A. niger* and *A. terreus*, respectively (Figure 8). 

## 4. Discussion

**Figure 9:**
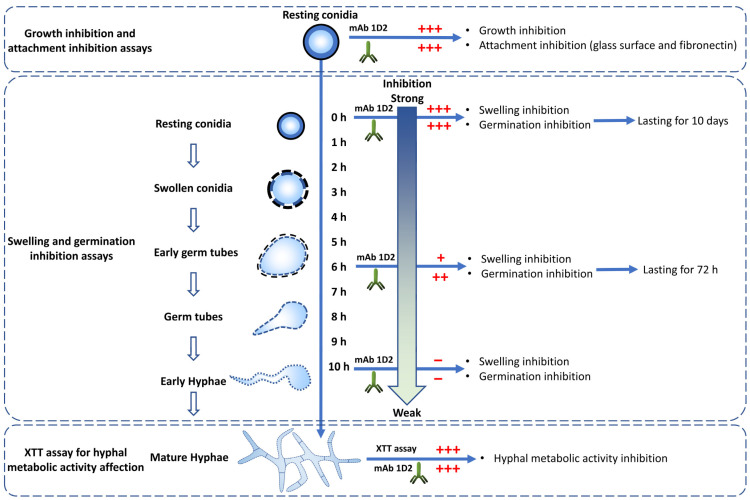
MAb 1D2 affects multiple important steps in the life cycle of *Aspergillus* species. MAb 1D2 interferes with the growth and attachment of resting conidia. In addition, it inhibits the swelling and germination of conidia. The earlier the antibody was added, the stronger the inhibition effect was. The mAb also showed damage on the filamentous hyphae metabolic activity. +++: strong inhibition; ++: moderate inhibition; +: mild inhibition; −: very weak inhibition.

In this study, we have demonstrated that the novel mAb 1D2 which binds specifically to a hyphal cell wall antigen is highly inhibitory to the growth of *A. fumigatus*, *A. flavus*, *A. niger* and *A. terreus*. All of these species can cause IA, but *A. fumigatus* is the most important. Detailed studies then demonstrated that 1D2 inhibited sequential steps in the pathogenesis of IA. Firstly, attachment of *Aspergillus* species to both an untreated surface and fibronectin-treated surface was strongly inhibited. Secondly, conidial swelling and germination were inhibited whether added to conidia with the initial inoculum or during incubation in SD media. Finally, mAb 1D2 damaged mature hyphae of these species (Figure 9). 

The inhibitory activity of the mAb 1D2 was initially demonstrated in dose escalation experiments, which found that the antibody inhibited growth of *A. fumigatus* ATTC strain and clinical strains and *A. flavus* at concentrations of 50 µg/mL in growth media. The strains of *A. terreus* and *A. niger* were inhibited less efficiently at this concentration. This might be caused by the binding of mAb 1D2 on the early swollen spores during incubation. It is possible that the reduced number of colonies may be due to conidial aggregation, because we could visually see some clusters in the tube for both mAb 1D2 and 6B10 groups when compared to the PBS group. In addition, Figure 2 shows decreasing CFUs of mAb 6B10 when the concentration was more than 6.25 µg/mL. This indicates that there may be a non-specific IgM-related aggregation. Each assay in this study was carried out using mAb 6B10 as an isotype control alongside a blank control (PBS). Compared to the mAb 6B10 group, the growth inhibition of mAb 1D2 was significantly higher than the mAb 6B10 group as shown in Figure 2, indicating that there is an additional effect of the 1D2 antibody. Moreover, we only observed the aggregation after the conidia were swollen. In the mAb 1D2 groups, we did not see aggregation before 12 h of incubation. Together, these results suggest that the mAb 1D2 has an inhibition effect on different *Aspergillus* growth, and this may be affected by the aggregation but is not the main cause. Subsequently, final working concentrations of 66 and 89 µg/mL were used in experiments designed to investigate the activity of 1D2 against key steps in the development and pathogenesis of *Aspergillus* infections. 

Adherence is the primary and pivotal first step for *Aspergillus* pathogens to invade the host tissues, and inhibition of adherence may determine the outcome of exposure to conidia. Sialic acid is one of the important molecules on the conidial wall which facilitates adhesion to the respiratory epithelial components such as fibronectin [31]. Many researchers have focused on composite material solid surface to mimic the attachment ability in the laboratory assays [16,27,32]. We used a similar approach and demonstrated that 1D2 inhibited the binding of conidia to both untreated and fibronectin-treated surfaces. Since apparent inhibition of binding can be caused by non-specific effects such as protein adsorption on the solid surface or membrane molecules, we have controlled for this by using 5% BSA as a “blocking” agent [33]. The difference of the adherence inhibition between BSA and mAb 6B10 was not significant, suggesting that the low level of inhibition found by mAb 6B10 was due to nonspecific surface adsorption and, like BSA, played a blocking role. Moreover, we reported that the resting spores started to swell within the first and second hour of incubation, and almost all were swollen by 8 h. Consequently, inhibition of adhesive induced by mAb 1D2 results from specific binding to *Aspergillus* antigens. Nevertheless, *Aspergillus* adhesion is a complicated process and involves various cell wall adhesin, secretory proteins and secreted enzymes [34,35].

Following adherence, conidia engulfed by the epithelial cells start to swell and develop germ tubes as a preliminary step for hyphae growth. As the filamentous hyphae are the main form of *Aspergillus* in lesions found in IA, preventing initial conidial swelling and germination may be the central limiting progression of IA [36]. MAb 1D2 suppressed the swelling and germination process of *A. fumigatus*, *A. flavus*, *A. niger* and *A. terreus* when compared to control mAb 6B10. This was apparent if mAb 1D2 was added within the first two hours, although the effect dropped sharply when added at later intervals. However, the inhibitory effect, once established, was long lasting, as it persisted for up to 10 days when incubated together with the *Aspergillus* conidia. This compares favourably with reports of inhibition from other antibodies which have reported effects lasting only a few hours [17,29,37]. The mAbs (3G11 and 5H5) lose their inhibition ability on germination when incubated for 10 h [17]. If conidia were incubated in growth media for 6 h before 1D2 was added, the conidia failed to develop germ tubes or hyphae over the next 72 h, but the conidia became progressively more swollen, suggesting that the conidia were surviving and that metabolic activity continued despite complete inhibition of hyphae formation. Together, these results indicate that inhibitory effects of 1D2 may persist so long as it is bound to the antigen and may inhibit germination of engulfed conidia in vivo.

The final stage of invasion is the development of invasive hyphae that are able to cross tissue boundaries and spread via the blood stream [24]. Hyphal growth begins very soon after gemination and proceeds rapidly [38]. To estimate the importance of the timing of exposure on overall germination and hyphae growth, changes in optical density at increasing intervals after addition of the antibody were measured. The formation of mycelia in the liquid culture media may be confounded by clumping of hyphae in the media that is mediated by glucan and glucosamine residues [39]. To overcome this, the *Aspergillus* samples were ultrasonicated to disperse hyphae within the media sample and at every indicated time points after the addition of mAb. As with the germination inhibition percentage assay, lower absorbance was detected in the culture media if mAb 1D2 was added at the first two hours of incubation, despite the increase in hyphae growth, after 2 h, there was about a 50% reduction in growth when the antibody was added 5 h after incubation started. 

To estimate the effect of 1D2 on mature hyphae, the XTT assay was performed on hyphae that had developed after overnight incubation of conidia. This assay was preferred to MTT, as it is more time-effective, has higher sensitivity and dynamic range, and measures cellular metabolic activity [40]. The studies demonstrated that there was 90% inhibition of metabolic activity in the ATCC strain of *A. fumigatus* and *A. flavus* with a little less activity against the clinical strains of *A. fumigatus*. We were unable to determine whether the hyphae were dead, as the antibody remained tightly bound despite washing, and the lack of growth may have been attributable to inhibition rather than killing. 

The results of these studies have several important implications. Firstly, 1D2 is a large IgM pentamer that we have shown binds to a large antigen (>37 kD) that is abundant in the hyphal cell wall [24]. The inhibition of adherence may result from steric hindrance, as IgM is a large molecule that could hinder access of surface antigens to adhesins including those on untreated slide surface and fibronectin-coated surface. Secondly, the inhibitory effect of 1D2 on *Aspergillus* species is long lasting, but is not lethal since the conidia swelled but did not germinate after incubation with mAb 1D2 for 10 days. It is not clear why this arrests the germination process, but it is possible that it interferes with cross linking of the structural components needed early in the generation of hyphae [41]. Thirdly, there is evidence that 1D2 inhibits the metabolism of established hyphae. Together, these results demonstrate that 1D2 will inhibit multiple important steps in the life cycle of pathogenic *Aspergillus* species. 

The experimental results stated in this research should be considered in light of some limitations. There is no direct evidence to show the binding of mAb and the antigens on the cell wall of *Aspergillus* or the secreted agents to play a therapeutic role in anti-*Aspergillus* infection in vitro. Additionally, possible changes in the morphology of hyphae when mAb 1D2 is added after the formation of hyphae have not been investigated. More studies of hyphal changes after the addition of the antibody at different times are needed. 

## 5. Conclusions

In conclusion, mAb 1D2 has promising growth suppression activity against the important invasive *Aspergillus* species in humans. Moreover, it inhibits the attachment of *Aspergillus* on a composited surface and fibronectin. Furthermore, mAb 1D2 has an inhibitory effect on *Aspergillus* conidia swelling and germination, predominantly in the first two hours of incubation. Finally, mAb 1D2 is able to inhibit the hyphae metabolic activity of *A. fumigatus*, *A. flavus*, *A. niger* and *A. terreus*.

The activity of 1D2 in vitro is sufficiently promising to justify animal studies to determine whether the effects can be replicated in vivo. In the first instance, the safety of parenterally administered antibody needs to be established. After that, there needs experiments designed to determine whether 1D2 has potential as a prophylactic agent when given to immune-suppressed animals before inoculation with conidia. These experiments should be designed initially to model the situation that patients requiring intensive chemotherapy are in that makes them vulnerable to IA. Subsequent experiments could then be performed to determine the value of 1D2 as a treatment of early IA. 

## Figures and Tables

**Table 1:** The diameters of the swollen conidia (*n* = 30) over time.

Time (h)	0	6	10	24	32	72
Diameter (median IQR)	2.37 (2.22–2.52)	5.45 (4.75–6.06) ^a^	5.66 (5.22–6.36) ^a^	6.42 (5.37–6.93) ^ab^	6.16 (5.60–8.62) ^abc^	10.78 (7.51–12.87) ^abcde^

Note: IRQ: interquartile range; ^a^: *p* < 0.001 versus 0 h group, ^b^: *p* < 0.01 versus 6 h group, ^c^: *p* < 0.01 versus 10 h group, ^d^: *p* < 0.001 versus 24 h group, ^e^: *p* < 0.001 versus 32 h group.

## Data Availability

All data generated or analysed during this study are included in this published article.
